# CUEDC2 Drives β-Catenin Nuclear Translocation and Promotes Triple-Negative Breast Cancer Tumorigenesis

**DOI:** 10.3390/cells11193067

**Published:** 2022-09-29

**Authors:** Shuyan Han, Huifeng Hao, Haibo Han, Dong Xue, Yanna Jiao, Yuntao Xie, Ye Xu, Longtao Huangfu, Jialei Fu, Shan Wang, Hong Sun, Pingping Li, Qun Zhou

**Affiliations:** 1 Department of Integration of Chinese and Western Medicine, Key Laboratory of Carcinogenesis and Translational Research (Ministry of Education), Peking University Cancer Hospital & Institute, Beijing 100142, China; 2 Department of Clinical Laboratory, Key Laboratory of Carcinogenesis and Translational Research (Ministry of Education), Peking University Cancer Hospital & Institute, Beijing 100142, China; 3 Familial & Hereditary Cancer Center, Key Laboratory of Carcinogenesis and Translational Research (Ministry of Education), Peking University Cancer Hospital & Institute, Beijing 100142, China; 4 Division of Gastrointestinal Cancer Translational Research Laboratory, Key Laboratory of Carcinogenesis and Translational Research (Ministry of Education), Peking University Cancer Hospital & Institute, Beijing 100142, China; 5 Department of Biochemistry and Molecular Biology, University of Maryland School of Medicine, Baltimore, MD 21201, USA

**Keywords:** triple-negative breast cancer (TNBC), CUEDC2, Wnt/β-catenin, nuclear translocation, competitive peptides

## Abstract

Hyperactivation of Wnt signaling is crucial in tumor formation. Fully elucidating the molecular details of how the cancer-specific Wnt signaling pathway is activated or contributes to tumorigenesis will help in determining future treatment strategies. Here, we aimed to explore the contribution of CUEDC2, a novel CUE-domain-containing protein, to the activation of Wnt signaling and the tumorigenesis of triple-negative breast cancer (TNBC) and to determine the underlying mechanisms. TNBC patient samples and disease-free survival (DFS) data were used to determine the association between CUEDC2 and TNBC progression. The effects of CUEDC2 on TNBC were examined in TNBC cells in vitro and in subcutaneous xenograft tumors in vivo. Gene knockdown, immunoprecipitation plus liquid chromatography–tandem mass spectrometry, pull-down, co-immunoprecipitation, localized surface plasmon resonance, and nuclear translocation analysis were used to uncover the mechanisms of CUEDC2 in regulating Wnt signaling and TNBC development. CUEDC2 is sufficient to maintain the hyperactivation of Wnt signaling required for TNBC tumorigenesis. The contribution of CUEDC2 plays a major role in determining the outcome of oncogenic Wnt signaling both in vitro and in vivo. Mechanistically, the CUE domain in CUEDC2 directly bound to the ARM (7–9) domain in β-catenin, promoted β-catenin nuclear translocation and enhanced the expression of β-catenin targeted genes. More importantly, an 11-amino-acid competitive peptide targeting the CUE domain in CUEDC2 blocked the interactions of CUEDC2 and β-catenin and abrogated the malignant phenotype of TNBC cells in vitro and in vivo. We observed that TNBC patients who exhibited higher levels of CUEDC2 showed marked hyperactivation of the Wnt signaling pathway and poor clinical outcomes, highlighting the clinical relevance of our findings. CUEDC2 promotes TNBC tumor growth by enhancing Wnt signaling through directly binding to β-catenin and accelerating its nuclear translocation. Targeting the interactions of CUEDC2 and β-catenin may be a valuable strategy for combating TNBC.

## 1. Introduction

Wnt/β-catenin signaling becomes dysregulated in many cancer types. Hyperactivation of Wnt signaling has been demonstrated as a critical regulator controlling tumor-initiating cell self-renewal and differentiation, tumor metastasis, and drug resistance to cancer therapy. The β-catenin-dependent signaling pathway is initiated through Wnt ligands binding to FZD and LRP/5/6, which disrupts the core complex, including AXIN, APC, and GSK3, leading to the stabilization of β-catenin. Subsequently, β-catenin is accumulated in the cytosol, translocated to the nuclear, and initiates the oncogenic transcription of its target genes [1]. β-catenin’s stability is tightly controlled by numerous other proteins. CK1α phosphorylates β-catenin at the N-terminus (S45/T41/S37/S33) and induces β-catenin degradation through E3 Ubiquitin Protein Ligase [2]. The picture of the protein-protein interaction for the maintenance of β-catenin’s stability in tumor cells is far from complete and remains under active investigation.

Triple-negative breast cancer (TNBC) is characterized by a lack of estrogen receptors (ER) and progesterone receptors (PR) or overexpressed human epidermal growth factor receptor 2 (HER2) [2]. TNBC accounts for 15–20% of all diagnosed breast cancers and it shows hyperactivation of Wnt signaling, which is a major driver of TNBC tumorigenesis. Hyperactivation of Wnt signaling in TNBC patients is unlikely due to *CTNBB1* mutations. Undefined mechanisms may contribute to the hyperactivation of Wnt signaling in TNBC tumor cells [3]. CUE domain containing 2 (CUEDC2) is a ubiquitously expressed small protein containing 287 amino acids [4]. CUEDC2 was first discovered to interact with PR and promote its degradation in a ubiquitin-dependent manner in breast cancer [5]. Consistent with these findings in breast cancer cells, CUEDC2 mediates the degradation of ER and contributes to the resistance of breast cancer to endocrine therapy [6]. Findings from the abovementioned studies suggest that the CUEDC2-mediated degradation of ER and PR could contribute to ER and PR silencing in TNBC. Further studies have revealed that CUEDC2 suppresses tumor progression in lung adenocarcinoma, glioma, acute myeloid leukemia, etc. [7,8,9]. CUEDC2 also promotes the development of ovarian serous carcinoma, hepatic cell carcinoma, colorectal cancer, etc. [10,11,12]. These findings highlight the different functions of CUEDC2 in various cancer types. Additionally, several studies have demonstrated that CUEDC2 carries out its functions by directly or indirectly interacting with some critical signaling targets, such as STAT3, NF-κB, and p38 MAPK, etc. [8,13,14]. As we identified that TNBC patients express remarkably high levels of CUEDC2, we hypothesized that CUEDC2 contributes to the hyperactivation of Wnt signaling in TNBC.

In the present study, we determined the impact of CUEDC2 on Wnt/β-catenin signaling in TNBC. We further examined the mechanisms by which CUEDC2 activates Wnt/β-catenin signaling in TNBC. To translate our findings into a new therapeutic strategy, we developed a small competitive peptide to abrogate the malignant phenotype of TNBC cells by disrupting the interactions of CUEDC2 and β-catenin, providing a novel therapeutic strategy for TNBC treatment.

## 2. Materials and Methods

### 2.1. TNBC Clinical Specimens

Primary TNBC specimens (*n* = 168 cases) containing tumors and matched adjacent normal tissues were obtained from the Peking University Cancer Hospital from patients who underwent surgery from 2003 to 2008 at the hospital. The median follow-up period was 3727.5 days. The following inclusion/exclusion criteria were applied: (a) pathological diagnosis of resectable stage I–III TNBC; (b) detailed follow-up information. Written and informed consent was obtained from all patients before the surgical operation and specimens’ collection. This project was approved by the ethical committee of the Peking University Cancer Hospital & Institute (2018KT98).

### 2.2. Tumor Xenograft Study

Here, 6–8-week-old NPG female mice (Vitalstar, Beijing, China) were randomized into groups (*n* = 5/group), and 2 × 10^6^ MDA-MB-231 cells with stable CUEDC2 knockdown (shRNA) in 100 μL of Matrigel / PBS were injected into the mammary pad of mice. Tumor growth was monitored twice a week, and they were finally harvested at 3–4 weeks after tumor cell injection. Animal studies were approved by the Peking University Cancer Hospital Animal Care Committee (EAEC 2018-23) and were carried out by following the institutional guidelines.

### 2.3. Cell lines and Cell Culture

The human TNBC cell lines MDA-MB-231 and MDA-MB-468 were obtained from ATCC (Manassas, VA, USA). HEK 293FT was purchased from the Chinese Academy of Medical Sciences & Peking Union Medical College (Beijing, China). Cells were cultured in DMEM high glucose medium with 10% fetal bovine serum (FBS), 100 U/mL penicillin, and 100 mg/mL streptomycin in a 5% CO_2_ humidified incubator at 37 °C.

For details of the methods of plasmid construction and cell transfection, the cell viability and plate colony formation assays, the cell migration and invasion assays, co-immunoprecipitation (co-ip) and IP-LC/MS/MS spectrometry, the pull-down assay, the establishment of the CUEDC2 and β-catenin interaction model, immunoblotting and immunohistochemistry, the peptide competition assay (PCA) and localized surface plasmon resonance (LSPR) analysis, and statistical analysis, please see the online supplementary materials and methods.

## 3. Results

### 3.1. Disease-Free Survival (DFS) Was Shorter in TNBC Patients Who Expressed Higher Levels of CUEDC2

**Figure 1:**
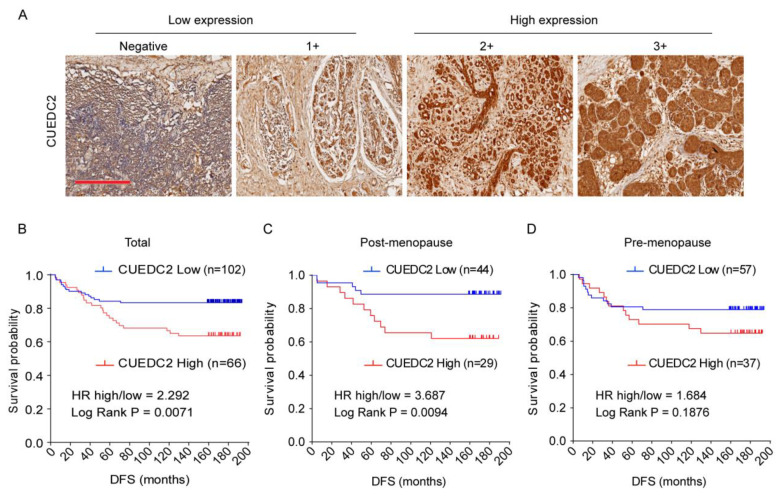
Expression of CUEDC2 in tumor tissues of TNBC. (**A**) IHC detection of CUEDC2 expression in tumor tissues, bar = 300 μm. (**B**–**D**). Kaplan-Meier (K-M) survival analysis on the disease-free survival (DFS) of the total (**B**), post-menopause (**C**), and pre-menopause (**D**) patients with different levels of CUEDC2 expression in tumor tissues.

CUEDC2 is responsible for resistance to estrogen therapy in ER-positive breast cancer [4,6]. However, it is still not clear whether CUEDC2 is involved in TNBC progression. To address the role of CUEDC2 in TNBC, we analyzed the association between CUEDC2 expression levels and clinical prognosis based on clinical data from 168 TNBC patients who received surgery at the Peking University Cancer Hospital between 2003 and 2008. CUEDC2 expression was characterized using standard immunohistochemistry and was determined by a pathologist in a blinded manner. Low and high expression levels of CUEDC2 were defined in tissue samples of TNBC patients (Figure 1A). The results showed that TNBC patients with higher expression of CUEDC2 in tumor tissues had a lower DFS probability (*p* = 0.0071, Figure 1B). We also analyzed the association between DFS and CUEDC2 expression in pre- and post-menopause patients separately. In the post-menopause subpopulation, DFS was significantly higher in the patients with lower expression of CUEDC2 (*p* = 0.0094, Figure 1C). However, the influences of CUEDC2 expression on DFS were largely attenuated in the pre-menopause TNBC patients (*p* = 0.1614, Figure 1D), suggesting that the primary effects of CUEDC2 on TNBC may be independent of female hormones.

### 3.2. CUEDC2 Affected the Phenotype and Tumor Growth of TNBC

**Figure 2:**
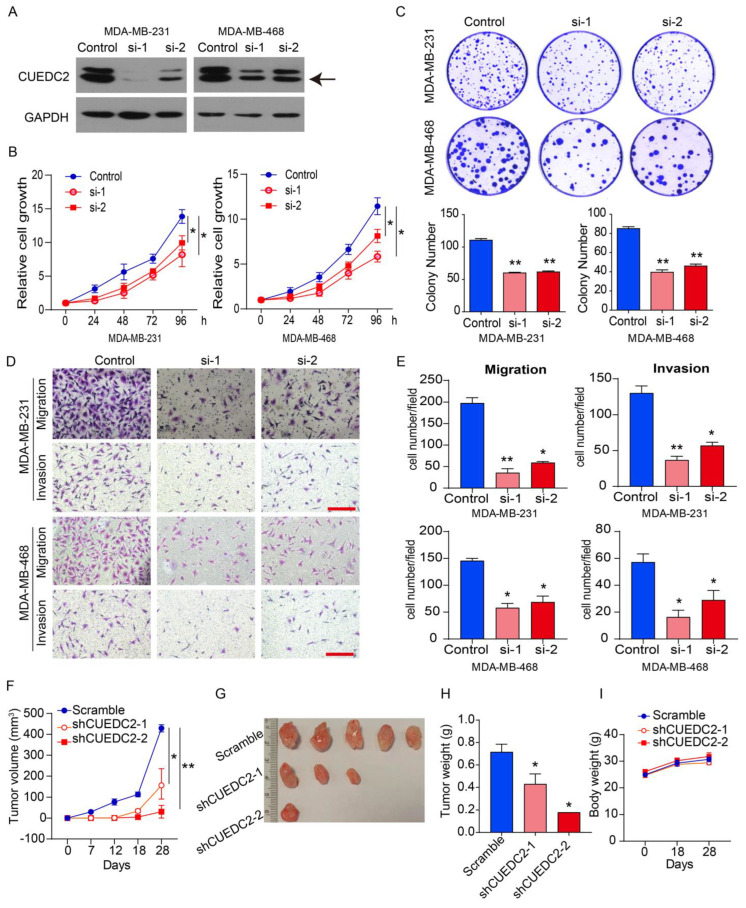
Knockdown of CUEDC2 suppressed malignant behaviors of TNBC cells in vitro and in vivo. (**A**) Western blot analyses of CUEDC2 as indicated. “Control” indicates that the cells were treated with negative RNA sequence. “si-1” and “si-2” denotes the two small interference RNA sequences targeting CUEDC2. Data are representative of three independent experiments. (**B**) Effects of CUEDC2 knockdown on cell viability of MDA-MB-231 and MDA-MB-468 cells. (**C**) Effects of CUEDC2 knockdown on colony formation ability of MDA-MB-231 and MDA-MB-468 cells. (**D**,**E**). Representative images (**D**) and statistical results (**E**) showing the effects of CUEDC2 knockdown on cell migration and invasion ability of MDA-MB-231 and MDA-MB-468 cells. Bar = 50 μm. (**F**) Effects of CUEDC2 shRNA knockdown in MDA-MB-231 cells on tumor growth in the mammary fatty pads of NPG mice. The scrambled shRNA served as a negative control. (**G**,**H**): Photos (**G**) and weights (**H**) of the tumors. (**I**) Average body weight for each group at different times. Statistical results are represented as mean ±S.E.M. The in vitro studies were repeated no less than three times. *n* = 5 in (**F**,**H**,**I**) * *p* < 0.05, ** *p* < 0.01 vs. control or scramble group.

As the clinical data suggested that CUEDC2 contributes to TNBC progression (Figure 1), we decided to examine the impact of CUEDC2 on the malignant behaviors of TNBC cells in vitro and in vivo. MDA-MB-231 (basal B subtype) and MDA-MB-468 (basal A subtype) cells are both TNBC tumor cells [15]. We depleted CUEDC2 expression in both cell lines using two small interference RNAs (Figure 2A). Knockdown of CUEDC2 suppressed cell proliferation, clonal formation, migration, and invasion abilities in MDA-MB-231 and MDA-MB-468 cells, highlighting that CUEDC2 promotes TNBC tumor cell growth and invasion in vitro (Figure 2B–E). Further, we inoculated the control and CUEDC2-knockdown TNBC cells into the mammary fatty pads of NPG mice and explored the role of CUEDC2 in TNBC tumor formation in vivo. The results showed that inhibiting CUEDC2 expression in tumor cells resulted in the remarkable suppression of tumor growth in vivo (Figure 2F–H) without obviously affecting the mice’s body weight (Figure 2I), emphasizing the role of CUEDC2 in modulating TNBC tumor formation in vivo.

To determine the impact of CUEDC2 on the phenotype of TNBC cells in vitro, CUEDC2 was overexpressed in MDA-MB-231 and MDA-MB-468 cells (Figure S1A). The results showed that the over-expression of CUEDC2 significantly enhanced the cell viability, colony formation, and cell migration and invasion of MDA-MB-231 and MDA-MB-468 cells (Figure S1B–E), further confirming the promotional effects of CUEDC2 on TNBC cell growth and invasion.

### 3.3. CUEDC2 Directly Binds to β-Catenin and Facilitates β-Catenin Nuclear Translocation

**Figure 3:**
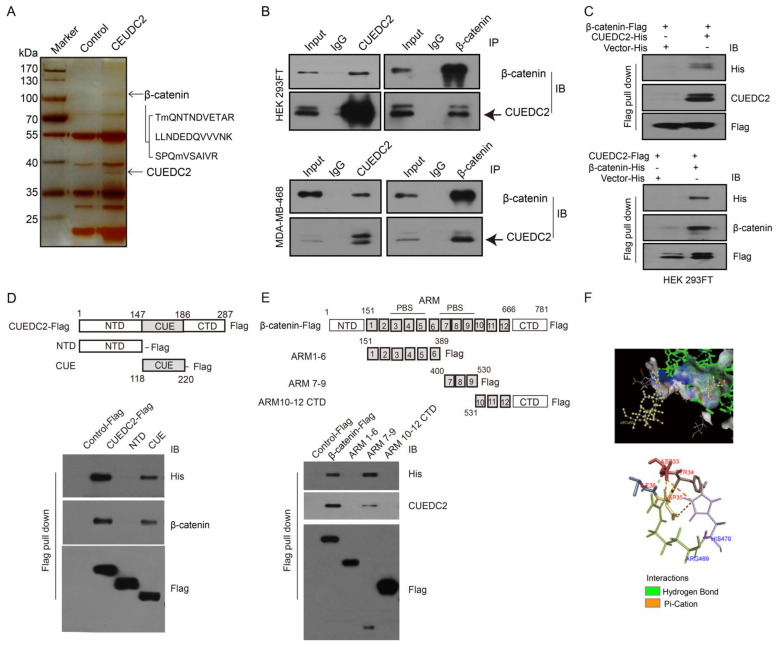
CUEDC2 directly interacts with β-catenin. (**A**) HEK-293FT cells were transfected with CUEDC2 or a control plasmid. Cell extracts were immunoprecipitated with an anti-CUEDC2 antibody. The immunoprecipitated proteins were analyzed using LC-MS/MS. Three peptide sequences of β-catenin were identified. (**B**) Upper panel: Exogenous Co-IP of CUEDC2 and β-catenin in the proteins from HEK-293FT cells with over-expressed CUEDC2 (**Left**) or β-catenin (**Right**). Lower panel: Endogenous Co-IP of CUEDC2 and β-catenin in the proteins from MDA-MB-468 cells with basal expression of CUEDC2 and β-catenin. (**C**) Flag pull-down was performed using purified β-catenin-Flag and CUEDC2-His (**up panel**) or CUEDC2-Flag and β-catenin-His (**low panel**), followed by immunoblotting (IB) with antibodies against His, CUEDC2, β-catenin or Flag. (**D**) Flag pull-down was performed using purified CUEDC2-Flag or Flag-labeled domains of CUEDC2 (NTD-Flag, CUE-Flag) and 6 × His-β-catenin, followed by IB with antibodies against His, β-catenin, or Flag. (**E**) Flag pull-down was performed using purified β-catenin-Flag or Flag-labeled domains of β-catenin (ARM1-6-Flag, ARM7-9-Flag, or ARM10-12 CTD-Flag) and 6×His-CUEDC2, followed by IB with antibodies against His, CUEDC2 or Flag. (**F**) Combination sites of CUEDC2 and β-catenin predicted by PyMOL. Data are representative of three independent experiments.

We next identified the molecular mechanism by which CUEDC2 accelerated TNBC tumor cell growth. Previous studies showed that CUEDC2 preferred to bind to a target protein for the modulation of signaling pathways [7,16,17,18]. As Wnt/β-catenin signaling is significantly involved in TNBC advancement, we hypothesized that CEUDC2 fosters TNBC cell growth through activating Wnt signaling. To test our hypothesis, we performed an IP-LC-MS/MS analysis and identified that CUEDC2 directly interacted with β-catenin protein (Figure 3A). The interaction between CUEDC2 and β-catenin was further confirmed using exogenous co-immunoprecipitation (Co-IP) in HEK-293FT cells and endogenous Co-IP in TNBC cells, respectively (Figure 3B). CUEDC2 and β-catenin were both detected in the proteins immunoprecipitated with antibodies against either CUEDC2 or β-catenin in the samples from HEK-293FT cells or MDA-MB-468 cells (Figure 3B). The flag pull-down assay using recombinant Flag-labeled β-catenin and His-labeled CUEDC2 or Flag-labeled CUEDC2 and His-labeled β-catenin revealed that CUEDC2 directly bound to β-catenin in vitro (Figure 3C). We used recombinant peptide segments of CUEDC2 or β-catenin to reveal that the binding of CUEDC2 with β-catenin occurred between the CUE domain of CUEDC2 and the ARM7-9 domain of β-catenin (Figure 3D,E). PyMOL also predicted that CUEDC2 might interact with β-catenin in its CUE domain at the ARG469 and HIS470 sites, and the main binding types are hydrogen bonds and pi-cation bonds (Figure 3F).

**Figure 4:**
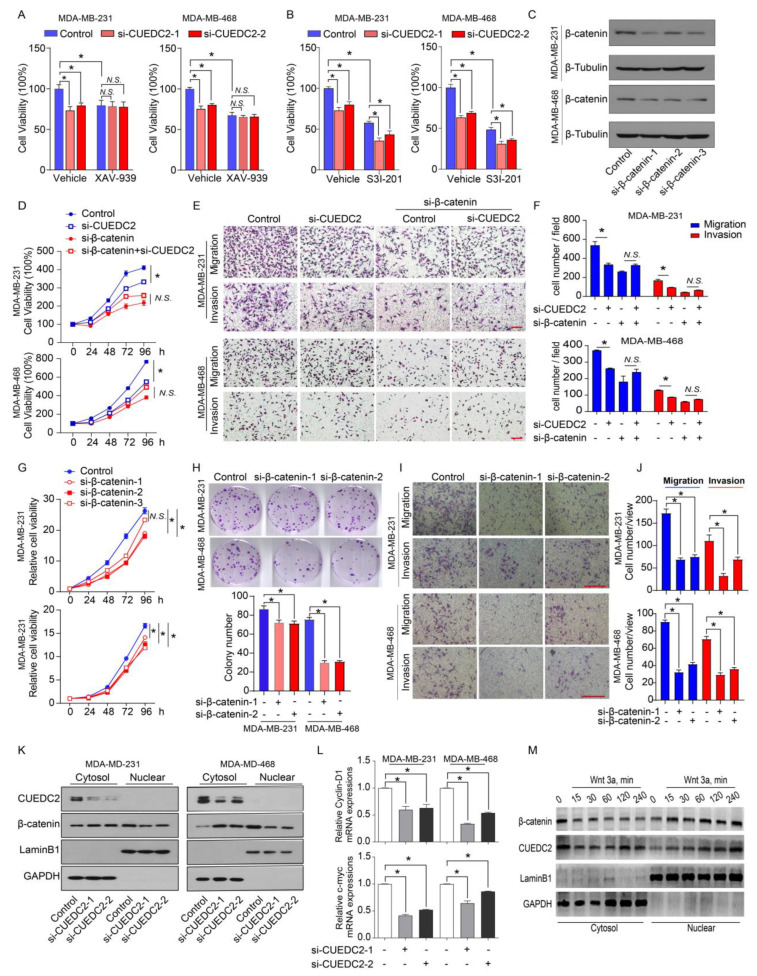
CUEDC2 promotes TNBC progression via interacting with β-catenin and accelerating its nuclear translocation. (**A**,**B**) Effects of CUEDC2 knockdown on proliferation of naïve, XAV-939-treated (**A**) or S3I-201-treated; (**B**) MDA-MB-231 cells and MDA-MB-468 cells. XAV-939, 1 μM, an inhibitor of β-catenin; S3I-201, 25 μM, an inhibitor of STAT3. (**C**) Western blot results show the efficiency of β-catenin knockdown in MDA-MB-231 and MDA-MB-468 cells. (**D**) Influences of β-catenin knockdown on the effects of CUEDC2 in modulating TNBC cell proliferation. The RNA sequences of si-CUEDC2-2 (si-2) and si-β-catenin-1 were used to analyze the interactions of CUEDC2-2 and β-catenin. (**E**,**F**) Representative images (**E**) and statistical results (**F**) showing the influences of β-catenin knockdown on the effects of CUEDC2 in modulating TNBC cell migration and invasion. (**G**–**J**) Effects of β-catenin knockdown on proliferation (**G**), clonal formation (**H**), migration and invasion (**I**,**J**) of MDA-MB-231 and MDA-MB-468 cells. (**K**) Effects of CUEDC2 knockdown on subcellular location of β-catenin in MDA-MB-231 and MDA-MB-468 cells. (**L**) Effects of knockdown of CUEDC2 on mRNA levels of c-myc and cyclin D1, two target genes of β-catenin in MDA-MB-231 cells. (**M**) Effects of Wnt 3a, a classical Wnt ligand, on nuclear translocation of β-catenin and CUEDC2. * *p* < 0.05, One-way ANOVA in (**A**,**B**,**F**,**H**,**J,L**); two-way ANOVA in (**D**,**G**). Data are representative of three independent experiments. Bar = 50 μm, “*N.S.*” means no significance.

Wnt-induced target genes represent major regulators of cancer stem cells responsible for the control of tumorigenesis. Since CUEDC2 directly interacts with β-catenin, we decided to determine if CUEDC2 functions through the activation of Wnt/β-catenin signaling. CUEDC2 knockdown significantly inhibited tumor cell proliferation in vitro and strongly reduced tumor formation in in vivo (Figure 2B,F). We then determined if the knockdown of CUEDC2 in tumor cells prevents cell proliferation through modulating the Wnt/β-catenin signaling pathway. Consistent with our observations in Figure 2B, CUEDC2 knockdown led to a reduction in tumor cell viability in both MDA-MB-231 and MDA-MB-468 cells (Figure 4A). XAV-939 stabilizes Axin, thereby enhancing the degradation of β-catenin, and serves as an inhibitor of β-catenin signaling [19]. Impressively, XAV-939 significantly inhibited cell growth (Figure 4A). However, CUEDC2 knockdown failed to reduce cell proliferation in XAV-939 treated tumor cells, suggesting that CUEDC2 inhibits tumor cell growth through Wnt/β-catenin signaling. The overexpression or constitutive activation of STAT3 promotes tumor cell proliferation [8,20], which is independent of CUEDC2 [21]. Although S3I-201, a STAT3 inhibitor, inhibited cell proliferation, CUEDC2 knockdown did not reduce cell proliferation in S3I-201 treated tumor cells, confirming that CUEDC2 inhibits tumor cell growth through a STAT3-independent manner (Figure 4B). We verified that XAV-939 and S3I-201 significantly inhibited β-catenin and STAT3 phosphorylation, respectively (Supplemental Figure S5). These data together demonstrate that the effect of the CUEDC2 knockdown on tumor cell growth was mediated by Wnt/ β-catenin signaling. To further determine whether β-catenin mediates the functions of CUEDC2 in tumor cell proliferation and invasion, endogenous β-catenin in tumor cells was also suppressed using siRNA strategies (Figure 4C). In control of MDA-MB-231 and MDA-MB-468 cells, siRNA CUEDC2 inhibited cell proliferation, migration, and invasion (Figure 4D–F). However, in the β-catenin knockdown TNBC cells, the effects of siRNA CUEDC2 on cell proliferation, migration, and invasion were diminished (Figure 4D–F), confirming that β-catenin is responsible for CUEDC2′s regulation of the tumor cell action. Furthermore, knockdown of β-catenin phenocopied the actions of CUEDC2 knockdown in TNBC cells (Figure 4G–J), which further validated the essential role of β-catenin in mediating the effects of CUEDC2 in accelerating the malignant behaviors of tumor cells.

Nuclear translocation is critical for β-catenin to promote tumorigenesis by initiating gene transcription [22]. As CUEDC2 binds to β-catenin and stabilizes it in tumor cells, we further explore the impact of CUEDC2 on the sub-cellular locations of β-catenin in control and CUEDC2 knockdown cells. Knockdown of CUEDC2 decreased the protein levels of β-catenin in total cells and nuclear; however, it strikingly increased the cytosol β-catenin proteins (Figure 4K and Figure S2), indicating a role of CUEDC2 in facilitating β-catenin’s nuclear translocation. Indeed, knockdown of CUEDC2 significantly reduced the expression of c-myc and cyclin D1, the downstream target genes of β-catenin (Figure 4L). Wnt3a is known to stabilize β-catenin and enhance its nuclear accumulation. Figure 4M shows that treatment with Wnt3a not only triggered the nuclear translocations of β-catenin but also induced CUEDC2 to enter the nuclear compartment in a time-dependent manner (Figure 4M and Figure S3). These data demonstrate that CUEDC2 promotes β-catenin’s nuclear translocation and β-catenin-mediated transcription in tumor cells.

### 3.4. Blocking of CUEDC2–β-Catenin Interaction by Competition Peptides (CP) Is a Novel Therapeutic Strategy for Treatment of TNBC

**Figure 5:**
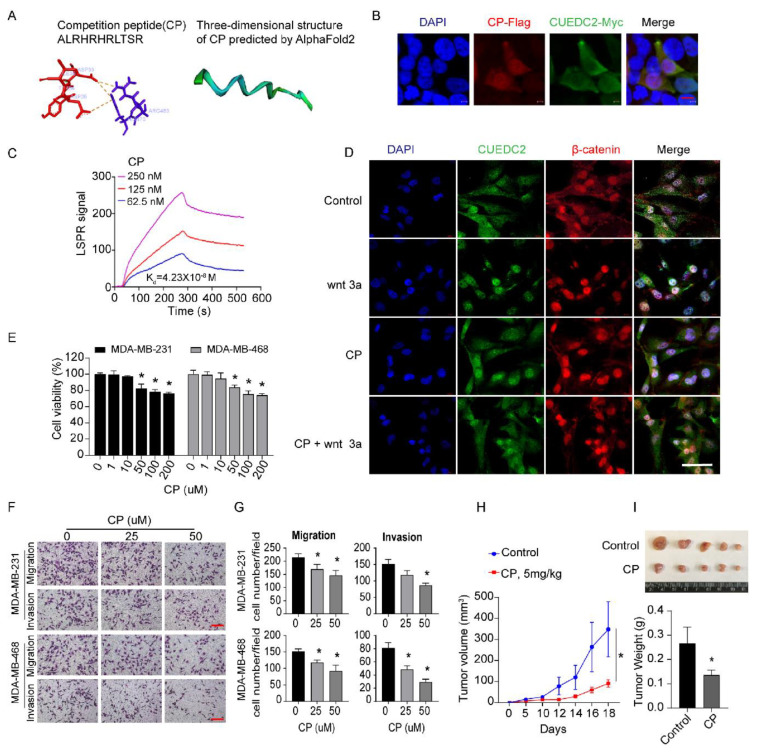
Interrupting CUEDC2-β-catenin interaction with competition peptides (CP) prevents malignant behaviors of TNBC in vitro and in vivo. (**A**) The amino acid sequence of CP and the predicted mode of combination between CP and CUEDC2 by AlphaFold2. (**B**) Co-localization of CP and CUEDC2 in HEK-293 FT cells. Bar = 10 μm. (**C**) LSPR analysis of the binding affinity of CP and CUEDC2. (**D**) Effects of CP (50 μM) on nuclear translocation of CUEDC2 (green) and β-catenin (red) induced by wnt 3a (100 ng/mL) in MDA-MB-231 cells. Bar = 50 μm. (**E**) Effects of CP on cell viability of MDA-MB-231 and MDA-MB-468 cells. (**F**,**G**) Representative images (**F**) and statistical results (**G**) showing the effects of CP on cell migration and invasion of MDA-MB-231 and MDA-MB-468 cells. Bar = 50 μm. (**H**) Curves of the tumor volume development in NPG mice treated with control and CP (5 mg/kg), respectively. (**I**) Photos and statistical results of tumor weight from the mice treated with Control or CP. * *p* < 0.05, vs. respective “0” or control; one-way ANOVA in (**E**,**G**); two-way ANOVA in (**H**); *t*-test in (**I**). The in vitro studies were repeated at least three times. *n* = 5 in (**H**,**I**).

Next, we examined whether disrupting the interactions of CUEDC2 and β-catenin was a promising approach to restrain TNBC tumor formation. Based on our findings (Figure 3E), we synthesized a short competition peptide of 11 amino acids from the sequence of the ARM domain in β-catenin (Figure 5A). PyMOL visualization showed that amino acid residues ASP33, TYR34, ASP35, and ILE36 from the CUE domain of CUEDC2 formed a pocket structure that could be directly bound by CP. As shown in Figure 5A, these docking sites formed strong binding interactions, mainly through pi–cation interactions. The three-dimensional structure of the CP scaffold was further analyzed via Alphafold2 (https://github.com/sokrypton/ColabFold, accessed on 27 July 2022) and is shown in Figure 5A. Immunofluorescent results showed the perfect co-localization of CP and CUEDC2 (Figure 5B). According to LSPR analysis, CP is effectively bound to CUEDC2 with a binding affinity (Kd) of 0.0423 μM (Figure 5C). In situ fluorescence staining revealed that CP treatment effectively blunted the remarkable nuclear accumulations of both CUEDC2 and β-catenin induced by Wnt3a, supporting the role of CP in blocking Wnt/β-catenin signaling (Figure 5D and Figure S4). Indeed, CP treatment significantly inhibited cell viability, cell migration, and invasion of TNBC cells (Figure 5E–G). More importantly, CP significantly suppressed the growth of TNBC tumors in the TNBC xenograft mouse model (Figure 5H,I), suggesting the therapeutic potential of targeting CUEDC2-β-catenin interactions in TNBC therapy.

### 3.5. Expression of CUEDC2 and β-Catenin was Positively Correlated in Clinical TNBC Tumors

**Figure 6:**
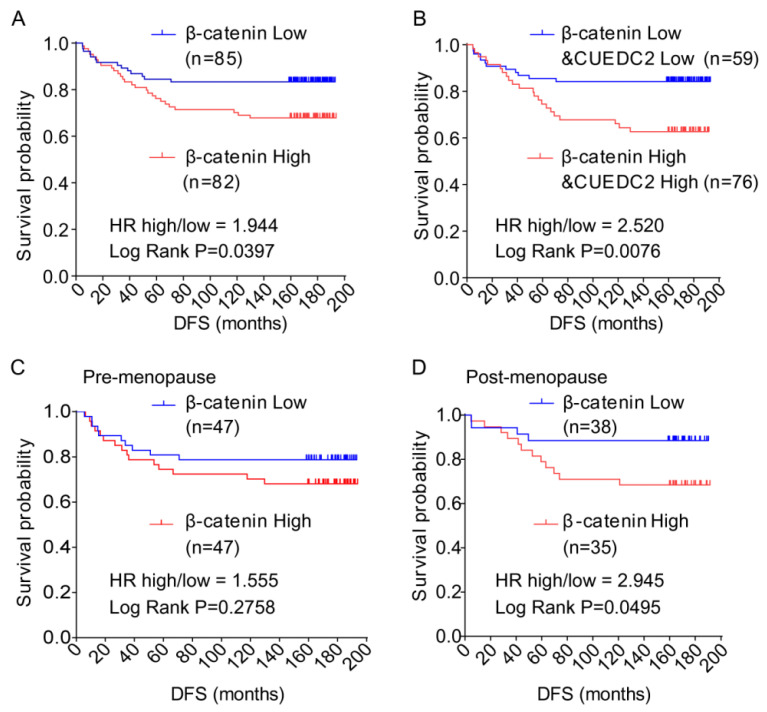
Disease-free survival (DFS) of TNBC patients with different levels of expression of β-catenin and β-catenin plus CUEDC2 in their tumor tissue. (**A**,**B**). K-M survival analysis on DFS of the TNBC patients with different levels of expression of β-catenin (**A**) and β-catenin plus CUEDC2 (**B**) in their tumor tissue. (**C**,**D**). K-M survival analysis on DFS of the post-menopause (**C**) and pre-menopause (**D**) patients with different levels of expression of β-catenin in their tumor tissue.

Finally, we aimed to confirm whether high levels of CUEDC2 and β-catenin correlate with poor prognosis for TNBC patients. We discovered that the CUEDC2 protein level is positively correlated with the β-catenin protein level in TNBC tumors (Table 1). In Figure 1, the clinical data show that DFS was shorter in TNBC patients with higher levels of CUEDC2. Here the results show that patients with higher levels of β-catenin in tumor tissues had significantly shorter DFS than those with lower levels of β-catenin (*p* = 0.0286, Figure 6A). We further observed that patients with higher levels of both β-catenin and CUEDC2 had even worse DFS than those with lower levels of both β-catenin and CUEDC2 (*p* = 0.0076, Figure 6B). Interestingly, when the status of menopause was considered, worse DFS was found in post-menopause patients with higher β-catenin expression (*p* = 0.0495, Figure 6C) but not in pre-menopause patients (Figure 6D). Together, this indicates the phenocopied trend of the association between CUEDC2 expression and DFS in total TNBC patients.

## 4. Discussion

Precise control of Wnt signaling is required to promote TNBC growth, progression, and the formation of metastasis. Aberrations in CUEDC2 gene expression have been reported in several human cancers; however, the role of CUEDC2 in the establishment of the hyperactivation of Wnt signaling in TNBC remains to be defined. We show here that CUEDC2 expression is negatively related to the DFS of TNBC patients. We further reveal that CUEDC2 promotes TNBC cell proliferation, migration, and invasion. Mechanistically, CUEDC2 accelerated Wnt/β-catenin signaling by directly binding to β-catenin and facilitating the nuclear translocation of β-catenin. A peptide targeting the binding sites between CUEDC2 and β-catenin disrupted the combination of CUEDC2 and β-catenin, and it suppressed the progression of TNBC in vitro and in vivo. Altogether, this study introduced a new role of CUEDC2 in TNBC, discovered its new interacting protein—β-catenin—and the novel action of CUEDC2 in regulating Wnt/β-catenin signaling, and presented a promising strategy for combating TNBC by targeting CUEDC2.

We found that an 11-amino-acid competitive peptide targeting the CUE domain in CUEDC2 blocked the interactions of CUEDC2 and β-catenin and abrogated the malignant phenotype of TNBC cells in vitro and in vivo. We did not test if the peptide can block the interaction between beta-catenin and other proteins such as Tcf/Lef. We are aware that the interaction between β-catenin and Lef extends along the armadillo repeats [23]; therefore, the peptide used by ours could block that interaction. We will test this possibility in our future studies.

It should be noted that CUEDC2 has multiple functions as an independent driver in different types of cancer [4]. Our clinical data overwhelmingly demonstrated that CUEDC2 promoted TNBC development. The clinical data, based on 168 TNBC cases, showed that patients with lower expression of CUEDC2 in tumor tissues had a much longer DFS after surgery. Using human-derived TNBC cells MDA-MB-231 and MDA-MB-468, we found that CUEDC2 positively regulated the malignant behavior of TNBC cells both in vitro and in vivo. To our knowledge, these are the first data exhibiting the impact of CUEDC2 in TNBC, the most aggressive type of breast cancer. A high expression level of CUEDC2 indicates a significant prognostic value for breast cancer patients [4]; thus, therapeutic drug-targeted CUEDC2 should be considered as a promising approach to eradicate Wnt signaling driving cell populations and, in consequence, TNBC. Indeed, peptides targeting the binding sites of CUEDC2 and β-catenin reduced the malignant phenotype of TNBC in vitro and caused the remarkable inhibition of tumor growth in vivo. Our present findings provide tangible means to treat TNBC by inhibiting the CUEDC2-mediated β-catenin signaling pathway.

The CUE domain in CUEDC2 binds to a target protein and subsequently degrades the target protein in a ubiquitin- or phosphorylation-dependent manner [5,6,7,18] CUEDC2 also serves as an inhibitor of anaphase-promoting complex/cyclosome-Cdh1 (APC/C^Cdh1^) or heat shock protein 70 (HSP70) by binding to APC/C^Cdh1^ or HSP70, respectively [16,24]. In our study, we revealed the novel action of CUEDC2. Instead of leading to the degradation or inhibition of an active protein, CUEDC2 directly bound to β-catenin, enhanced β-catenin nuclear translocation, and promoted the transcription of β-catenin downstream target genes (Figure 4). When CUEDC2 was knocked down using shRNA, the total protein levels of β-catenin were decreased, and we observed reduced transcription of the β-catenin gene. However, to our surprise, the cytosol levels of β-catenin proteins were not decreased but strikingly increased (Figure 4K), suggesting a pivotal role of CUEDC2 in promoting the cellular distribution of β-catenin. As is known, nuclear β-catenin initiates oncogenic signals and is responsible for most of the malignant behaviors of cancer. Thus, our study demonstrates that CUEDC2 promotes the hyperactivation of Wnt signaling by modulating the processes of β-catenin’s nuclear translocation.

## 5. Conclusions

The present study has revealed a significant role of CUEDC2 in promoting TNBC. CUEDC2 fulfills its function by binding to β-catenin and facilitating β-catenin’s nuclear translocation. Targeting the CUEDC2-β-catenin interaction with a small peptide could effectively suppress TNBC development as well as its malignant phenotype (Figure S5). Our findings are valuable for the further recognition of the roles of CUEDC2 in breast cancer. Additionally, our study provides a new target for TNBC; thus, it is meaningful for the development of new therapeutic approaches for TNBC based on targeting CUEDC2 and/or β-catenin.

## Figures and Tables

**Table 1:** Correlation of CUEDC2 and β-catenin protein levels in TNBC tumor tissues.

		CUEDC2				Total	Pearson Correlation	
		0.00	1.00	2.00	3.00			
**β-catenin**	0.00	71	0	2	0	73	*r*	0.804
	1.00	1	4	5	0	10	*p*	0.000 **
	2.00	1	15	34	0	50		
	3.00	2	8	24	1	35		
Total		75	27	65	1	168		

** means significant correlation at ≤0.01 level (bilateral).

## Data Availability

The datasets used and/or analyzed during the current study are available from the corresponding author upon reasonable request.

## Supplementary Materials

The following supporting information can be downloaded at: https://www.mdpi.com/article/10.3390/cells11193067/s1, Figure S1: Overexpression of CUEDC2 enhanced malignant behaviors of TNBC cells in vitro.; Figure S2: Effects of CUEDC2 knock down on β-catenin protein expressions.; Figure S3: Statistical results for Figure 4M; Figure S4: Statistical results for Figure 5D; Figure S5: Effects of XAV-939 on β-catenin protein levels (A) and effects of S3I-201 on STAT3 phosphorylation(B).
