# Supplementary material for: CUEDC2 Drives β-Catenin Nuclear Translocation and Promotes Triple-Negative Breast Cancer Tumorigenesis
Source: Cells. 2022 Sep 29;11(19):3067. doi: 10.3390/cells11193067 (PMC9563079; doi:10.3390/cells11193067)
Supplement: Supplementary file 1 [file cells-11-03067-s001.zip › cells-1937861-supplementary.pdf]

Supplementary materials and methods

Supplementary methods

TNBC clinical specimens

Primary TNBC specimens (n=168 cases) containing tumors and matched adjacent normal tissues were obtained from the Peking University Cancer Hospital from patients who underwent surgery from 2003 to 2008 at the hospital. The median follow-up period was 3727.5 days. The following inclusion/exclusion criteria were applied: (a) pathological diagnosis of resectable stage I-III TNBC; (b) detailed follow-up information. Written and informed consent was obtained from all patients before the surgical operation and speci-mens' collection. This project was approved by the ethical committee of the Peking Uni-versity Cancer Hospital & Institute (2018KT98).

Tumor xenograft study

Here, 6-8-week old NPG female mice (Vitalstar, Beijing, China) were randomized into groups (n= 5/group), and 2×10^6 MDA-MB-231 cells with stable CUEDC2 knockdown (shRNA) in 100 μl of Matrigel / PBS were injected into the mammary pads of mice. Tumor growth was monitored twice a week, and they were finally harvested at 3-4 weeks after tumor cell injection. Animal studies were approved by the Peking University Cancer Hospital Animal Care Committee (EAEC 2018-23) and were carried out by following the institutional guidelines.

Cell lines and cell culture

MDA-MB-468 and MDA-MB-231 cells, which respectively represents the basal A and B subtype breast cancer cells, are classical TNBC cells, and were obtained from ATCC (Manassas, Virginia, United States). HEK 293FT was purchased from the Chinese Academy of Medical Sciences & Peking Union Medical College (Beijing, China). Cells were cultured in DMEM high glucose medium with 10% fetal bovine serum (FBS), 100 U/mL penicillin, and 100 mg/mL streptomycin in a 5% CO2 humidified incubator at 37°C.

Plasmid construction and cell transfection

CUEDC2 (NM_024040), CTNNB1 (NM_001904, gene name of β-catenin) and domain deletion sequences were cloned into a pcDNA3.1 (+) expression vector with Flag or His Tag. Plasmids were transfected into TNBC cells, and selected by 500 μg/ml G418 solution for 2 weeks to obtain stable overexpressing cell line. The lentivirus RNAi shuttle vector containing the sequence for short-hairpin RNA (shRNA) for CUEDC2 or CTNNB1 were transfected into HEK 293FT cells together with the lentiviral helper plasmids PLP1, PLP2 and pLP/VSVG to generate lentiviruses. The lentiviruses infected TNBC cells were screened with 1 μg/ml blasticidin S to obtain stable knockdown expression cells.

Cell viability and plate colony formation assay

MDA-MB-231 and MDA-MB-468 cells were both used for determination of the cell viability and the plate colony formation under similar protocols. The cell viability was examined with a Cell Counting Kit-8 (CCK-8, Dojindo). Briefly, a total of 5×10^3 cells/well for intervention and 2×10^3 cells/well for growth curve determination were seeded into 96-well plates, treated and cultured for indicated periods of time. Then, before determination of the cell viability, the medium of the cells were changed into serum-free DMEM medium containing CCK-8 (the ratio of DMEM to CCK-8 is 10:1). The cells were incubated with the DMEM-CCK8 mixture for 1-4 hours. Finally, the cell viability were quantified by measuring the absorbance value of the DMEM-CCK8 mixture at the wavelength of 450 nm using a microplate reader (Tecan, Männedorf, Switzerland). For plate colony formation assay, 200 cells/well were cultured in 60 mm dishes for 2 weeks. The colony more than 50 cells were counted after fixation with 4% formaldehyde for 30 min and staining with 0.1% crystal violet for 30 min at room temperature.

Cell migration and invasion assays

To examine the effects of CUEDC2 on cell migration, the MDA-MB-231 and MDA-MB-468 cells were firstly subjected to CUEDC2 knockdown for 48 hours. Thereafter, the cells were treated with 10 μg/ml of mitomycin-C (Sigma) for 1 h at 37°C to inhibit cell division. Then, the cells (1×10^4) were added into the upper chamber of a Transwell insert (8.0 μm pore polycarbonate membrane) in 100 μL of DMEM high glucose

medium without FBS. 500 μL of DMEM with 10% FBS was added into the lower chamber as a chemoattractant. Twenty-four hours later, the ability of cell migration was determined by photographing the cells migrated outside of the upper chamber. In detail, the cells attached on the upper chamber were fixed with 4% formaldehyde for 30 min, followed by staining with 1% crystal violet for 30 min at room temperature. Then, the cells inside the upper chamber were removed using a cotton swab, and the remaining migrated cells outside the upper chamber were photographed in five randomly selected microscopic fields. The resulting pictures were finally analyzed automatically using the software of Image-Pro-Plus 6.0 (IPP, Media Cybernetics, Maryland, USA). The process for determining cell invasion is same to that for examining cell migration except that the upper chambers for cell invasion were pre-coated with Matrigel (DMEM : Matrigel = 1:8) at 37 ℃ for 1 h.

Co-immunoprecipitation (Co-IP) and IP- LC/MS/MS Spectrometry

For endogenic Co-IP, the cellular protein extracts were incubated overnight with the appropriate primary antibodies or normal immunoglobin G (IgG) as control at 4°C. For exogenic Co-IP, HEK-293FT cells were transfected with Flag-CUEDC2 or Flag-CTNNB1 or control plasmids for 48 h, and the cellular protein were extracted. Then, protein A/G or anti-FLAG M2 magnetic beads were added and incubated for 4 h at 4°C. The beads were washed and the immune complexes were subjected to SDS-PAGE followed by mass spectrometry analysis or immunoblotting with antibodies.

Pull down assay

HEK-293FT cells were transfected with Flag-CUEDC2, Flag-CTNNB1, 6 × His-CUEDC2, 6 × His-CTNNB1 or control plasmid for 48 h, respectively. Scraped cells were lysed with RIPA. Then, the cellular protein of Flag-CUEDC2 was incubated with anti-FLAG M2 magnetic beads for 3 h at 4°C and washed three times with PBS. The beads were incubated with lysates containing protein 6 × His-CTNNB1 or control plasmid for 3 h at 4°C, separately. Finally, the bound proteins were detected by Western blotting. The procedures for Flag-CTNNB1 and 6× His-CUEDC2 are the same as described above.

Establishment of CUEDC2 and β-catenin interaction model

The three-dimensional structure of protein domains was obtained from PROTEIN DATA BANK (PDB, https://www.rcsb.org/). The CUE domain of the human CUE domain containing protein family (PDB access number: 2DHY) contains 67 amino acids and the armadillo repeat region of β-catenin (PDB access number: 2Z6H) contains 644 amino acids. Both structures were confirmed by X-RAY diffraction. The molecular docking was carried out using Autodock and Vina to construct the interaction model between CUE domain and β-catenin. Docking poses generated by *autogrid* programs can be directly loaded into PyMOL through the Autodock/Vina-plugin. The possibility to visualize different binding site properties provides valuable insights for the next structure-based drug design.

Immunoblotting and immunohistochemistry

Cells were lysed with radioimmunoprecipitation assay (RIPA) buffer containing a protease inhibitor cocktail (Roche, Basel, Switzerland). Protein concentration was assessed with a BCA protein assay kit (Thermo Fisher Scientific, Waltham, Massachusetts, USA). Equal amounts of protein for each sample (20 μg) were separated on 10% SDS-PAGE gels, and subsequently transferred to PVDF membranes (Millipore, Merck, Darmstadt, Germany). The membranes were blocked for 1 h in TBST containing 5% nonfat milk and then incubated overnight with the primary antibodies at 4°C. The membranes were then incubated with HRP-conjugated secondary antibodies and the protein bands were visualized by ECL reagents (Millipore, Merck, Darmstadt, Germany). Densitometry analyses were performed by Image J software. The primary antibodies used are as follows: CUEDC2 (ab79036, Abcam, Cambridge, UK), β-catenin (610154, BD pharmingen, Franklin Lake, New Jersey, USA), GAPDH (AC033, Abclonal, Wuhan, China), β-Tubulin (AC021, Abclonal, Wuhan, China), Lamin B1 (TDY049C, TDY Biotech, Beijing, China), His (AE003, Abclonal, Wuhan, China), Flag (AE005, Abclonal, Wuhan, China). For analyzation, each Western blot study was performed for at least three times, and the representative images were shown in the main manuscript.

Paraffin sections were subjected to antigen retrieval and a staining protocol with Dako EnVision™$^{+}$System-HRP DAB. Antibody of CUEDC2 or β-catenin (1:100 dilution) was added to the sections, which were incubated overnight at 4°C. The sections shown for comparison in each figure were stained at the same time and photographed under

identical conditions. The case is defined as positive if more than 10% of the tumor cells are stained. Expressions of CUEDC2 were categorized into 1 +, 2 + and 3 + by a pathological expert in Peking University Cancer Hospital, in a blind manner. The negative and 1 + categories were defined as low expressions of CUEDC2. While, the 2 + and 3 + categories were defined as high expressions of CUEDC2.

Peptide competition assay (PCA) and localized surface plasmon resonance (LSPR) analysis

A competition peptide (CP) was designed based on protein–protein molecular docking result and was synthesized by Shanghai Apeptide CO, LTD. (Shanghai, China). CP, labeled with Biotin, was synthesized to examine the interaction between CP and CUEDC2. The equilibrium dissociation constant (Kd) of peptide for CUEDC2 was determined by OpenSPR (Nicoya, Canada) according the manufacture's protocol. In brief, Biotin-labeled peptide (1 mg/ml) was fixed on Biotin sensor chip after adding Streptavidin. Then, the purified CUEDC2 (ab171602, Abcam, Cambridge, UK) was continuously diluted into different concentration and injected into chamber from low to high concentration (62.5~250 nM). The kinetic parameters were calculated by Trace Drawer software. The three-dimensional structure of the designed competitive peptide scaffold was predicted by AlphaFold (https://github.com/sokrypton/ColabFold).

Statistical analysis

Data were analyzed with SPSS 26 and GraphPad Prism 8. Chi-Square test was used to

analyze the CUEDC2 expression and the clinical pathological characteristics. Kaplan-Meier method was used to analyze the survival. The continuous variables with normal distribution and equal variance (F-test) between/within the groups are expressed as the mean ± S.E.M. The statistical significance was determined using a two-tailed Student's t-test between two groups, one-way ANOVA with Bonferroni post hoc tests for multiple comparisons. *P*<0.05 was considered as statistically significant difference.

Supplemental figures and figure legends

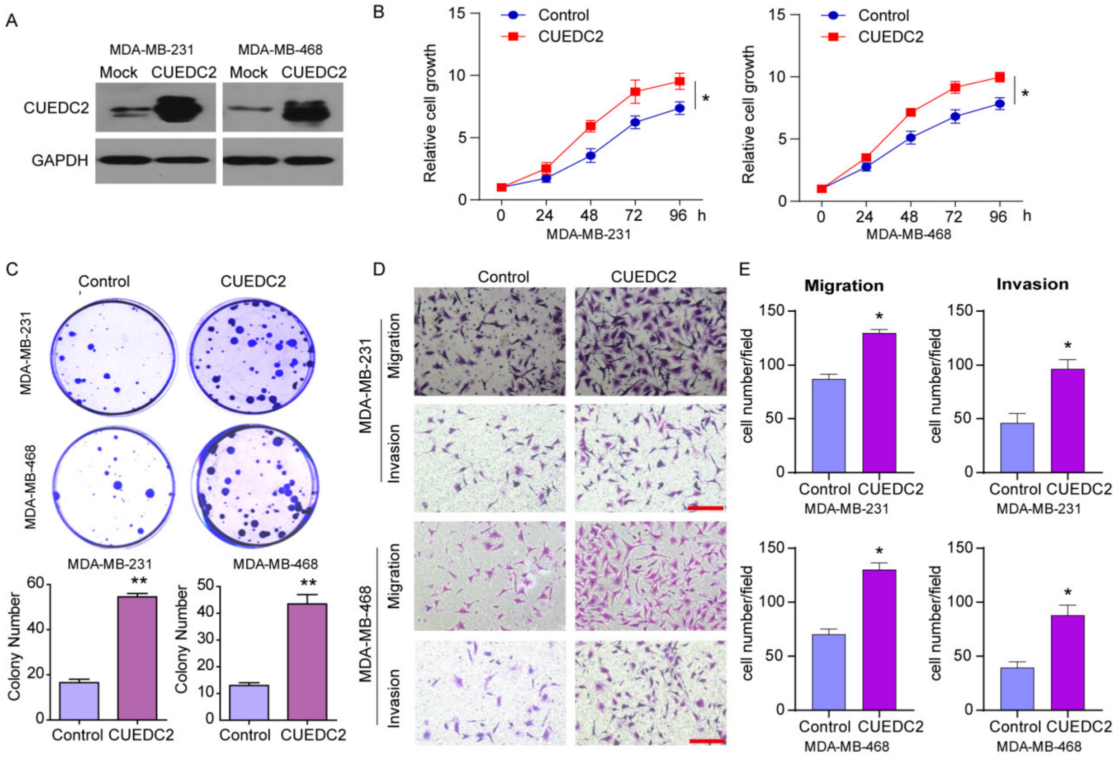

Supplemental Figure S1. Overexpression of CUEDC2 enhanced malignant behaviors of TNBC cells *in vitro*. A. Western blotting was used to examine the expression levels of CUEDC2. **B.** Effects of CUEDC2 overexpression on cell viability

of MDA-MB-231 and MDA-MB-468 cells. **C.** Effects of CUEDC2 overexpression on cell colony formation of MDA-MB-231 and MDA-MB-468 cells. **D&E.** Representative images (**D**) and statistical results (**E**) showing the effects of CUEDC2 over-expression on cell migration and invasion ability of MDA-MB-231 and MDA-MB-468 cells. Bar=50 μm. Data in B, C and E are represented as mean ± S.E.M, and were analyzed using two-way ANOVA (B) or t-test (C&E). **P*<0.05, ***P*<0.01. Each experiment was repeated three times.

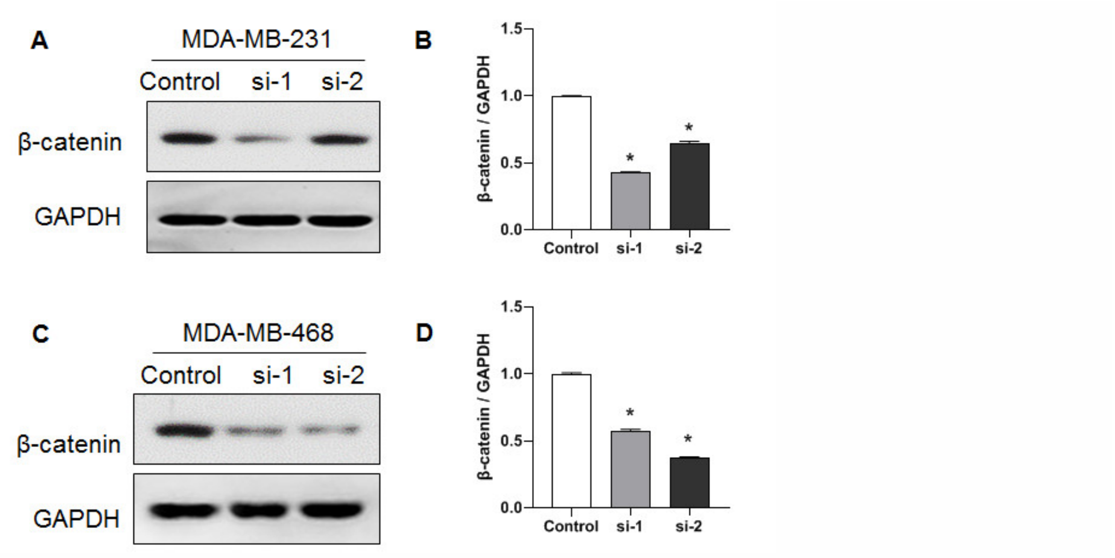

Supplemental Figure S2. Effects of CUEDC2 knock down on β-catenin protein expressions. A&C. Representative images showing the effects of CUEDC2 knock down on β-catenin protein expressions in MAD-MB-231 (**A**) and MAD-MB-468 (**C**) cells. Western blots were quantified. **B&D.** Statistical results showing the effects of CUEDC2 knock down on β-catenin protein expressions in MAD-MB-231 (**B**) and MAD-MB-468 (**D**) cells. si-1, si-2 represents si-CUEDC2-1 and si-CUEDC2-2, respectively. n=3; *p<0.05

v.s. Control. One-way ANOVA with Turkey post-hoc tests.

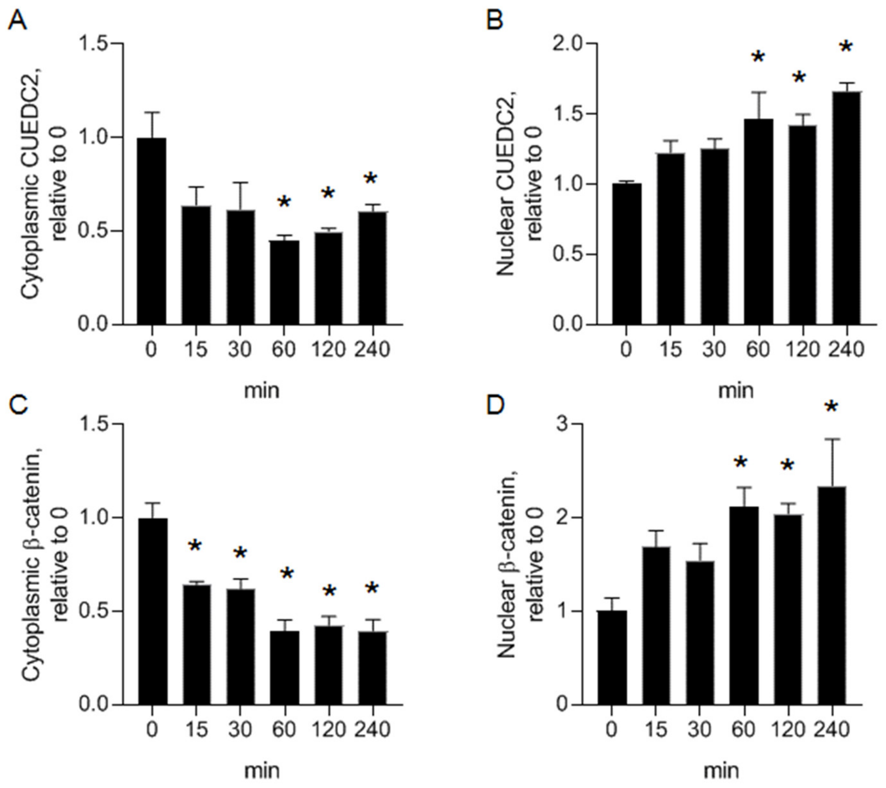

Supplemental Figure S3. Statistical results for Figure 4M. A&C. Wnt 3a (100 ng/mL) treatment decreased cytoplasmic CUEDC2 (**A**) and β-catenin (**C**) in MAD-MB-231 cells. **B&D.** Wnt 3a treatment increased nuclear CUEDC2 (**B**) and β-catenin (**D**) in MAD-MB-231 cells. n=3; *p<0.05 *v.s.* 0 min; One-way ANOVA with post hoc tests.

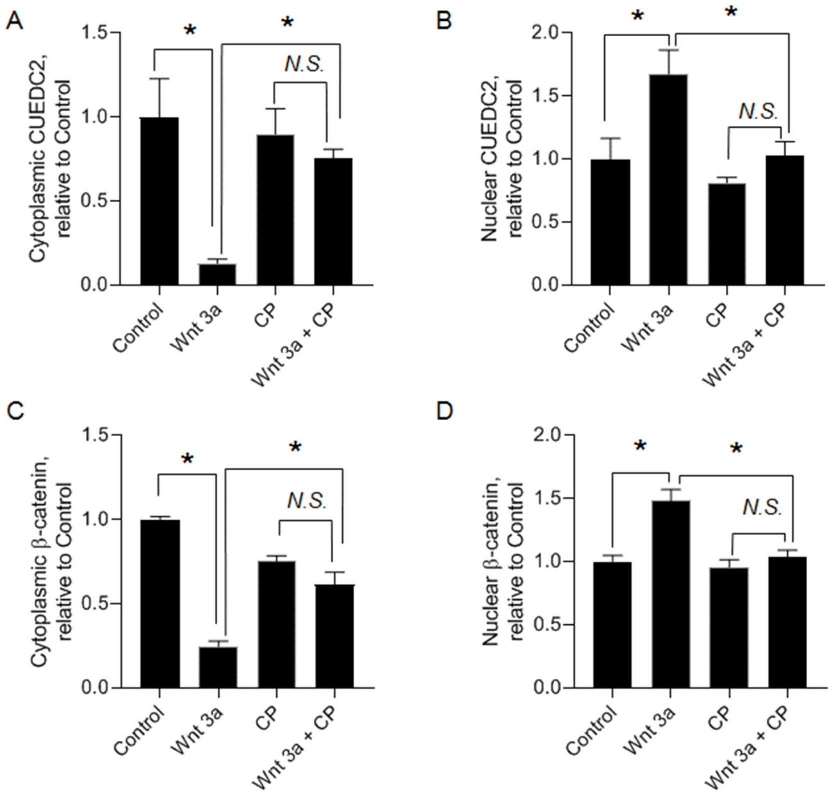

Supplemental Figure S4. Statistical results for Figure 5D. A&C. Statistical results of cytoplasmic CUEDC2 (**A**) and β-catenin (**C**) under treatment of Wnt 3a (100 ng/mL), CP (50 μM) and CP plus Wnt 3a in MAD-MB-231 cells. **B&D.** Statistical results of nuclear CUEDC2 (**B**) and β-catenin (**D**) under treatment of Wnt 3a (100 ng/mL), CP (50 μM) and CP plus Wnt 3a in MAD-MB-231 cells. n=3; *p<0.05 *v.s.* Control; One-way ANOVA with post hoc tests.

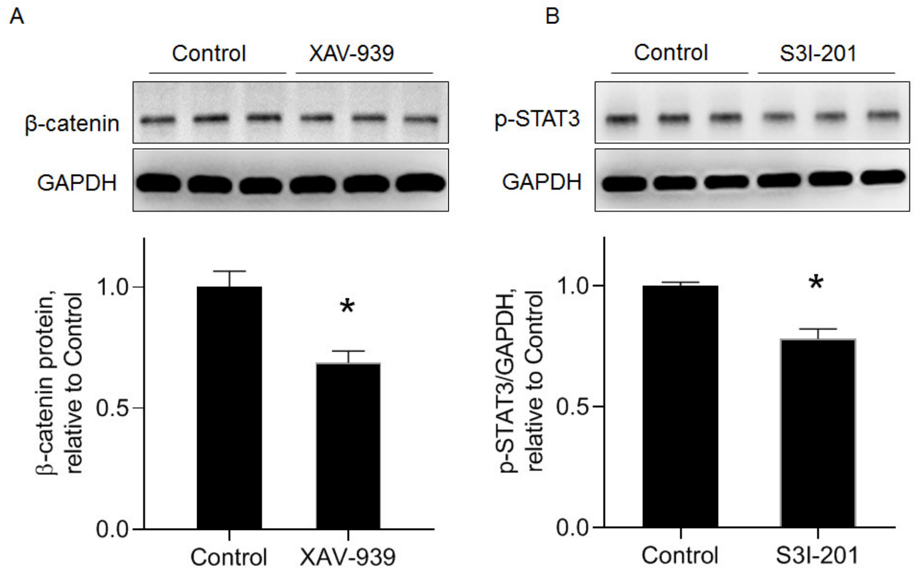

Supplemental Figure S5. Effects of XAV-939 on β-catenin protein levels (A) and effects of S3I-201 on STAT3 phosphorylation (B). MDA-MB-468 cells were treated with 1 μM XAV-939 and 25 μM S3I-201 for 12 hours. Western blots were performed and quantified as indicated. n=3, *p<0.05 *v.s.* control.
